# High-Flow Nasal Cannula Therapy as an Adjuvant Therapy for Respiratory Support during Endoscopic Techniques: A Narrative Review

**DOI:** 10.3390/jcm13010081

**Published:** 2023-12-22

**Authors:** Marta Corral-Blanco, Javier Sayas-Catalán, Ana Hernández-Voth, Laura Rey-Terrón, Victoria Villena-Garrido

**Affiliations:** Avenue de Cordoba, s/n, 28041 Madrid, Spainvvillena@separ.es (V.V.-G.)

**Keywords:** high flow nasal cannula, acute hypoxemic respiratory failure, hypoxemia, endoscopy, bronchoscopy, airway intervention, sedation

## Abstract

High-flow nasal cannula (HFNC) is a respiratory support technique that delivers a controlled concentration of oxygen with high flow, heat, and humidity via the nasal pathway. As it has many physiological effects, its use has increased for a variety of clinical indications; however, there is limited guidance on using HFNC as a respiratory support tool during endoscopic procedures. We conducted a narrative review to evaluate the effect of HFNC as an adjuvant tool during fiberoptic bronchoscopy (FOB), upper gastrointestinal tract endoscopy, and surgical procedures in adults. A search of the PubMed and Cochrane databases were performed. Approximately 384 publications were retrieved, and 99 were selected (93 original works and 6 case reports with a literature review). In patients who underwent FOB, HFNC appears to be superior to conventional oxygen therapy (COT) in preventing hypoxaemia. In contrast, for gastrointestinal endoscopy, the current evidence is insufficient to recommend HFNC over COT in a cost-effective manner. Finally, in surgical procedures such as laryngeal microsurgery or thoracic surgery, HFNC has been shown to be a safe and effective alternative to orotracheal intubation. As the results are heterogeneous, we advocate for the need for more quality studies to understand the effectiveness of HFNC during endoscopic procedures.

## 1. Introduction

High-flow nasal cannula (HFNC) is a modern technique for respiratory support that delivers a controlled concentration of oxygen with high flow, heat, and humidity via the nasal pathway [1]. Since HFNC has various physiological effects [2], it has become more commonly used for various clinical indications, ranging from non-invasive respiratory support in acute respiratory failure (ARF) [3] to a tool for weaning or respiratory support in high-risk patients. HFNC has gained popularity as a non-invasive form of respiratory support in acute healthcare settings. Nevertheless, evidence supporting its efficacy has only recently surfaced. The specified guidelines propose evidence-based recommendations for using HFNC in conjunction with other non-invasive respiratory support methods in adults with acute respiratory failure (ARF) [4]. However, there is limited guidance on using HFNC as a respiratory support tool during endoscopic procedures.

During bronchoscopy, certain physiological challenges may confer a heightened risk for the development of respiratory failure [5]:

- Sedation, necessary for patient toleration of the technique, may induce respiratory depression, requiring airway interventions such as mandibular traction, oropharyngeal airway device insertion, non-invasive ventilation (NIV), and even orotracheal intubation (OTI) and invasive mechanical ventilation.
- Other circumstances that arise during fiberoptic bronchoscopy (FOB) include:
  - The effective airway diameter is reduced when advancing the bronchoscope beyond the glottis; suctioning during FOB can result in alveolar recruitment loss and atelectasis formation.
  - Instillation of local anesthetics or saline solution during certain procedures, such as bronchoalveolar lavage (BAL), may cause alveolar flooding and affect the ventilation/perfusion ratio [6,7,8,9].

HFNC has been proposed as an advantageous oxygen delivery modality over conventional oxygen therapy (COT) during endoscopic procedures [10]. Because of its physiological effects [2], it is able to compensate for the patient’s inspiratory demands during the test, decrease anatomical dead space by achieving a washout of exhaled air, reduce resistance generated by the upper airways, and generate a mild positive expiratory pressure that allows greater lung recruitment, preventing the formation of atelectasis. The main drawback with respect to COT is the larger calibre of the high-flow cannula, which can make nasal access during FOB difficult.

On the other hand, NIV has previously been used as a respiratory support tool during FOB [6]. However, the emergence of HFNC as the simplest device has increased the use of this alternative support tool. The aim of the present narrative review is to examine the published evidence on the use of HFNC as an adjuvant tool during FOB, upper gastrointestinal tract endoscopy, and surgical procedures, including its advantages and pitfalls.

## 2. Materials and Methods

A search in the PubMed and Cochrane databases were performed by 30 May 2023, with this strategy: ("Endoscopy" [Mesh] OR endoscop* OR Bronchoscop* OR laryngoscop* OR mediastinoscop* OR thoracoscop*) AND “high flow."

The search was limited to English-language papers, studies made on humans and adults, and between 2003 and 2023.

Exclusion criteria were related to animal studies, paediatric population, and high flow, not to upper airway or respiratory tract interventions.

A review of the selected papers was performed in batches by each of the authors and, later on, summarised and discussed by the whole group.

According to the PRISMA recommendations checklist [11], the following items were not fulfilled:

- Title: Do not consider it a systematic review, as our intention was to perform a narrative review;
- No risk bias assessment nor effect measurements were performed, as we did not intend to perform a meta-analysis.

The results will be presented in a narrative way, explaining and interpreting published evidence.

## 3. Search Results and Main Findings of Included Trials

**Figure 1:**
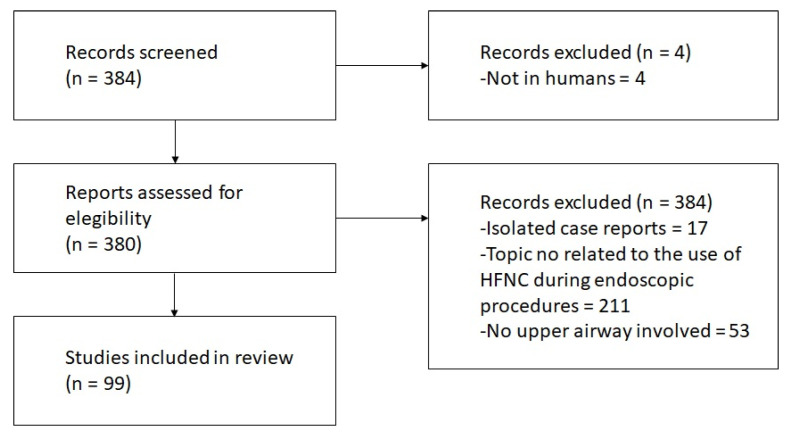
PRISMA-based flowchart for the selection of studies. HFNC: High-Flow Nasal Cannula.

Approximately 384 publications were retrieved, and 99 of them were selected, which included 93 original works and 6 case reports with a literature review. A total of 4 papers were excluded as not being performed in humans, 53 papers were not related to the upper airway (for example, “high flow spinal fluid leak”), 211 papers did not express the use of HFNC during the procedure, and 17 were isolated case reports with no further analysis. Figure 1 shows the PRISMA-based flowchart for the selection of studies.

The main findings of the included trials are presented in Table 1.

## 4. HFNC in FOB

FOB is a fundamental diagnostic and therapeutic procedure in the assessment of the airways and lung parenchyma. It is an interventional technique that, like all procedures, can be associated with complications, including the development of hypoxaemia [6,7,8,10,11,27,28]. Hypoxaemia can also lead to serious cardiac events such as atrial or ventricular arrhythmias [8]. The occurrence of any of these complications may require interruption of the technique to access the airway and ensure adequate ventilation, reversal of anaesthesia, or prolongation of the procedure [8].

For this reason, adequate oxygen monitoring during the procedure by at least continuous pulse oximetry is essential, as is having a source of supplemental oxygen available in case hypoxaemia develops [27,28]. In recent years, an increasing number of clinical trials and meta-analyses have attempted to analyse the advantages and disadvantages of different devices that can be used for oxygen supplementation during FOB, including COT (with nasal cannula and oxygen mask with or without reservoir), HFNC, continuous positive airway pressure (CPAP), and NIV [6,7,8,10,11].

### 4.1. HFNC vs. Other Oxygen Supply Systems during FOB

#### 4.1.1. HFNC vs. COT

COT consists of administering a certain flow rate of 100% oxygen, which is mixed with ambient air until the patient’s inspiratory flow is complete. Its main advantage is that it is accessible, inexpensive, and easy to use, but its drawback is that the FiO_2_ administered is variable and depends on the patient’s breathing pattern [6,10].

During FOB, COT via nasal cannula or simple oxygen mask has been shown to be effective in preventing desaturation episodes in patients with pre-procedural baseline oxygen saturation <93%, a history of chronic obstructive pulmonary disease (COPD), or during certain procedures such as FOB with BAL or bronchial brushing [6,29].

However, several studies and meta-analyses have shown that HFNC is superior to COT in improving oxygen saturation, reducing episodes of desaturation [6,7,8,10,11,12,13], reducing procedure interruptions [8,10,13], and avoiding additional airway manoeuvres [10,13]. This benefit is greater the lower the baseline oxygen level [6]; no effect was found on hypercapnia [7,8,10,11] or on the incidence of OTI [11].

During FOB with BAL, HFNC also appears superior to COT in preventing worsening oxygen saturation in both ambulatory patients [6,14] and hypoxaemic patients admitted to intensive care units [30,31], with no difference found in the rate of OTI [30].

#### 4.1.2. HFNC vs. CPAP/NIV

CPAP systems generate continuous positive airway pressure, which reduces airway resistance and allows the recruitment of atelectatic lung areas [6]. Its main drawback is poor interface tolerance and the need for special masks with accessory ports for bronchoscope passage [28]. This system has been compared with COT in the randomised control trial (RCT) by Maitre et al. [32], with CPAP showing a higher oxygen saturation value during and after the procedure, with less need for ventilator support in the 6 h after FOB.

On the other hand, NIV has the advantages of CPAP, adding the beneficial effects of pressure support, which decreases the respiratory effort generated by the patient and ensures adequate ventilation in situations of deeper sedation. As a drawback, in addition to mask-related discomfort, it is more difficult to achieve adequate patient-ventilator interaction due to the presence of asynchronies caused by leaks and airway manipulation during the procedure [6,7]. NIV has been effectively applied during FOB in patients with respiratory failure, improving PaO_2_/FiO_2_ compared to patients treated with COT [33].

When comparing the two systems with respect to HFNC, an improvement in oxygenation has been observed with NIV/CPAP [6,7,15], especially in the most hypoxaemic patients [34]. Also, desaturation episodes below 90% were lower with NIV/CPAP [6,34]. Other meta-analyses found no difference in desaturation episodes [7]. On the other hand, Saksitthichok et al. [34] found greater dyspnoea after FOB in patients treated with NIV vs. HFNC. Therefore, although the results are contradictory, in patients with more severe hypoxaemia CPAP/NIV seems superior to HFNC. It also seems sensible that during procedures with deeper sedation, an NIV or even a laryngeal mask should be used to ensure adequate ventilation and gas exchange [6].

### 4.2. HFNC in Special Procedures and Situations

In special populations such as lung transplant patients or in certain techniques such as endobronchial ultrasound (EBUS) or foreign body removal, HFNC also appears superior to COT in preventing the development of hypoxaemia.

Ben-Menachem et al. [35] evaluated HFNC vs. COT during FOB with transbronchial lung biopsy in lung transplant patients, observing that HFNC allowed a reduction in the number of mild and moderate desaturations, also reducing test interruptions.

EBUS procedures pose an increased risk for the development of hypoxaemia during the technique due to the larger calibre of the bronchoscope and balloon inflation [8]. The efficacy of HFNC compared to COT has been evaluated in studies such as Irfan et al. [36] and Douglas et al. [16], where higher oxygen saturation during the test and decreased episodes of desaturation were observed.

For rigid bronchoscope foreign body removal, Abdel et al. [37] compared HFNC with apnoeic oxygenation, observing superior oxygenation in patients treated with HFNC and lower end-expiratory carbon dioxide after the procedure.

## 5. HFNC in Upper Gastrointestinal Endoscopy

### 5.1. HFNC vs. COT in Hypoxemia Prevention

Endoscopic upper gastrointestinal procedures are performed under sedation, with hypoxia being one of the most frequent complications arising from these procedures [38]. The advantage that HFNC could provide over COT is to prevent these episodes of desaturation as it can provide high and constant oxygen flows [39].

Several RCTs [17,40,41] and meta-analyses [42,43,44] conclude that HFNC reduces the incidence of hypoxaemia and consequently the need for airway interventions. However, these data are not consistent. Khanna et al. [45], in a recent meta-analysis comparing HFNC vs. COT during the upper gastrointestinal endoscopic procedure under sedation, state that the evidence for improved oxygenation and decreased incidence of endoscopic procedure interruption is of low quality due to a lack of significant direct correspondence in terms of differences in population and HFNC schedules. In the same vein, Lee et al. [46] in a meta-analysis do not demonstrate that HFNC reduces the overall incidence of hypoxaemia, explaining the discrepancies in the results between the different studies by the different statistical models used by each systematic review.

Finally, RCT in both low-risk hypoxia patients [47] and high-risk patients [18], defined as patients with body mass index (BMI) > 30 or diagnosed with obstructive sleep apnoea, or assessed as ASA (American Society of Anesthesiologists physical status class) III or IV, found no advantage of HFNC over COT in controlling hypoxaemia.

### 5.2. HFNC and Hyperoxia Management

Hyperoxia increases pulmonary oxidative stress, reduces alveolar surfactant levels, increases microcirculatory vasoconstriction, and may cause resorptive lung atelectasis by increasing the shunt effect [48]. For this reason, Zhang et al. [19] conducted a RCT evaluating optimal FiO2 with HFNC (FiO_2_ 50% vs. 100%) in elderly patients, concluding that HFNC reduces hypoxia vs. COT without finding significant differences in hypoxaemia or adverse events with HFNC when FiO_2_ was 50% or 100%.

Sawase et al. [20] propose a further step in the control of hyperoxia by proposing a study in patients undergoing endoscopic retrograde cholangiopancreatography (ERCP) under moderate sedation with HFNC without oxygen supplementation. They conclude that there is no significant difference in the incidence of hypoxaemia between COT and HFNC. This suggests, according to the authors, that the positive pressure and dead space reduction achieved by HFNC may be effective in maintaining oxygen saturation without requiring additional FiO_2_.

### 5.3. HFNC vs. COT in Hypercapnia Management

The high airflow used with HFNC has the beneficial effect of dead space lavage, which would facilitate both oxygenation and carbon dioxide removal under sedation [39]. However, in most studies with HFNC in upper gastrointestinal endoscopy, the carbon dioxide level has not been shown to be different between the HFNC vs. COT groups at the end of the endoscopic procedure [17,47].

In the study by Sawasse et al. [20], although a time trend in carbon dioxide washout was observed with HFNC, no differences in hypoxaemia or hypercapnia were demonstrated between the two groups that might indicate an improvement in gas exchange at the end of the procedure.

In a post hoc analysis of the study by Mazzefi [17], in the group of at-risk patients with a diagnosis of COPD, it was observed that patients treated with HFNC during endoscopy under sedation had a significantly higher incidence of hypercapnia, showing no improvement in hypoxaemia control at the end of the endoscopic procedure compared to the group treated with COT.

### 5.4. HFNC vs. COT in a High-Risk Population

Conclusive data on the benefit of HFNC vs. COT in controlling hypoxaemia in upper gastrointestinal endoscopy under sedation may have been inconclusive because the different populations studied were highly heterogeneous. Therefore, studies have been conducted in an attempt to clarify the benefits for certain risk groups.

In the elderly, HFNC has been shown to be effective in controlling hypoxaemia vs. COT in both gastroscopy [19] and ERCP [49]. ERCP is usually performed with the patient in a prone position to facilitate the procedure, and sedation is usually deeper, which increases the risk of hypoxaemia and hypoventilation. In an RCT comparing HFNC vs. COT in elderly patients undergoing prone ERCP, HFNC demonstrated better control of hypoxaemia and decreased need for procedure interruption [21].

In patients defined as high-risk, obese patients with a BMI > 30, diagnosed with obstructive sleep apnoea or with ASA III or IV, there was no significant difference in the reduction of hypoxaemia or in the need for upper airway manoeuvres in patients treated with HFNC vs. those treated with COT in a post hoc study of an RCT [17], in subgroup studies of a meta-analysis [44], or in an RCT performed in this risk group [47].

## 6. HFNC in Surgical Procedures

### 6.1. Laryngeal Surgery

Surgical procedures performed over the airway pose a potential difficulty in ensuring patient ventilation during the procedure as the anaesthetist and surgeon must share the working space. Therefore, anaesthetic techniques that allow good exposure of the area while maintaining adequate oxygenation and ventilation are indispensable.

To accomplish this, there are several non-OTI techniques that can be used, such as supraglottic or subglottic jet ventilation or apnoea oxygenation with HFNC. HFNC may be useful in certain surgical procedures in low-risk (non-obese) patients, ensuring that it is possible to “rescue” the patient with supportive techniques such as jet ventilation or OTI in cases of decreased oxygen saturation.

Supraglottic jet ventilation is advocated over HFNC by some authors as being able to maintain a more stable oxygen saturation throughout the surgical procedure [22], but it also has disadvantages such as the risk of airway barotrauma, potential dissection or trauma of the supraglottis, and mucosal edoema [23].

The use of HFNC for spontaneous ventilation during surgery has been given several names: “STRIVE Hi” (SponTaneous Respiration using IntraVEnous anaesthesia and High-flow nasal oxygen) [24,50] or “NIDP” (non-intubated deep paralysis) [51]. Its advantages are due to the maintenance of good oxygenation due to its high flow and FiO_2_, which allows for uninterrupted surgery [52], upper airway lavage that helps maintain normal carbon dioxide levels, and the provision of warm, humidified air that more closely approximates physiological ventilation. Another important benefit of HFNC over OTI is that the duration of general anaesthesia in procedures with muscular paralysis and OTI is much longer than the duration of the surgery itself, especially in chordal microsurgery, where surgery can last even minutes, making the overall procedure inefficient [51].

HFNC has been shown to be a feasible and effective technique in laryngeal surgery during procedures that may be more prolonged [25,53,54]. Studies now show that apnoea techniques are safe and as effective as IOT in laryngeal microsurgery [55]. Recently, HFNC has also been tested in endoscopic surgical procedures of the hypopharynx, such as the correction of Zenker’s diverticulum, cricopharyngeal hypertrophy, and other upper esophageal disorders, with good results, allowing both intervention and recovery to be shorter [56].

### 6.2. Thoracic Surgery

In thoracic surgery, complete lobar resection has traditionally been considered the gold standard of treatment; however, anatomical segmentectomy has gained popularity in patients with compromised cardiopulmonary function as well as small and peripheral lung nodules [57].

Segmental resection by video-assisted thoracoscopic surgery (VATS), both multiportal and uniportal, has been commonly performed under general anaesthesia and OTI. Recent publications demonstrate that a less invasive anaesthetic technique during VATS for anatomical segmental resections in patients treated with HFNC is possible and safe [58], resulting in shorter anaesthetic induction time, shorter operative time, less intraoperative bleeding, and a shorter hospital stay [24]. It could even prevent intraoperative hypothermia, which is associated with bleeding and postoperative cardiac complications, especially in elderly patients [26].

Face masks and nasal oxygen cannula are the traditional and classic form of oxygen therapy, but new devices such as supraglottic ventilation, or HFNC, have demonstrated certain advantages over COT: supraglottic devices prevent upper airway obstruction caused by relaxation of the pharyngeal musculature during surgical anaesthesia, while HFNC has been associated with less post-surgical atelectasis [59]. However, when HFNC is used in VATS, there may also be some decreased muscle tone with reduced upper airway calibre and facial muscle relaxation with oral opening, which may decrease the efficacy of HFNC compared to supraglottic ventilation devices [22].

## 7. Conclusions

Despite advances in the understanding of the physiological effects and the extension of the use of HFNC beyond ARF, its role and efficacy during endoscopic procedures are not entirely clear.

For FOB, HFNC appears to be superior to COT in preventing hypoxaemia, especially in patients with deeper preoperative desaturation. However, when HFNC is compared with CPAP or NIV, the latter devices appear to be superior to HFNC in patients with more severe hypoxaemia or who require deeper sedation during the procedure.

In contrast, for gastrointestinal endoscopy under sedation, we believe that the current evidence is insufficient to make a recommendation in favour of the use of HFNC over COT in a cost-effective manner. We recommend close monitoring during the procedure to avoid hypoxaemia and hyperoxia, with appropriate titration of oxygen delivered.

In surgical procedures such as laryngeal microsurgery or thoracic surgery (i.e., VATS segmentectomy), HFNC could be a safe and effective alternative to OTI. However, jet ventilation seems to allow a more stable saturation to be maintained during the procedure with better control of the upper airway, although this procedure is not free of complications.

As the results are heterogeneous over mixed populations, with high variability in titration and parameters of HFNC and huge variations in sedation procedures, we advocate for the need for more quality studies that allow us to better understand the role of HFNC and select patients who may benefit the most.

Finally, as a limitation of this study, it should be pointed out that only PubMed and Cochrane databases were used in the search, and other databases may contain some articles that could provide additional information on the subject.

## Figures and Tables

**Table 1:** The main findings of the included trials.

References Author, Year	Study Design/Sample Size	Population and Type of Intervention	Sedation Methods	Respiratory Support Type	Main Results Summary
Arias-Sanchez, 2023 [12]	SC 40	Adult patients in acute care with a presumptive diagnosis of pneumonia receiving oxygen and undergoing FOB	Dexmedetomidine, Hydrochloride, Topical Anesthesia (Lidocaine 2%)	HFNC: Flow 60 lpm, Tª 35 °C, and FiO_2_ 40% vs. COT: FiO_2_ 40% through a nasal cannula	HFNC vs. COT has a smaller decrease in SpO_2_ levels during the procedure (94% vs. 90%; *p* = 0.40); and less variation between SpO_2_ before FOB and the lowest SpO_2_ during FOB: 2% vs. 4.5%; *p* = 0.01.
Zhang, 2022 [13]	RCT 176	Adults at risk of hypoxemia with a STOP-BANG score ≥ 3 during FOB under deep sedation	Propofol, Opioids	HFNC: Flow 60 lpm and FiO_2_ 100% vs. COT: Flow 6 lpm through a tight-fitting facemask	HFNC vs. COT reduces the incidence of oxygen desaturation (4.6% vs. 29.2%; *p* < 0.001) and the airway intervention (5.7% vs. 48.3%; *p* < 0.001).
Longhini, 2022 [14]	RCT 36	Adult outpatients undergoing FOB with BAL	Topical anaesthesia	HFNC: Flow 60 lpm, Tª 31 °C, and FiO2 21% if SpO_2_ > 95% or set to reach SpO_2_ > 95% vs. COT to maintain SpO_2_ > 94%.	HFNC improves gas exchange (56% of desaturation with COT vs. 11% with HFNC; *p* < 0.001), avoids loss of end-expiratory lung volume, and prevents an increase in diaphragm activation.
Simon, 2014 [15]	RCT 40	Critically ill patients with acute hypoxaemic respiratory failure undergoing FOB	Propofol, Topical anaesthesia (lidocaine 0.8%)	HFNC: Flow 50 lpm vs. NIV: PEEP 3–10 cm H_2_O and IPAP 15–20 cm H_2_O. Full face mask.	NIV was superior to HFNC regarding oxygenation before, during, and after FOB in patients with moderate to severe hypoxaemia
Douglas, 2018 [16]	RCT 60	Adults undergoing EBUS with conscious sedation	Propofol, Midazolam, Opioids, Topical 2% lidocaine	HFNC: Flow 30–70 lpm and FiO_2_ 100% vs. COT at 10–15 lpm via a bite block	Higher SpO_2_ after pre-oxygenation and during the procedure with HFNC vs. COT (100% vs. 98% and 97.5 vs. 92%, respectively; *p* < 0.001).
Mazzeffi, 2021 [17]	RCT 262	Adults with moderate to high risk for hypoxemia during advanced EGD	Propofol, Fentanyl or Midazolam	HFNC: Flow at 20 lpm vs. COT at 6 lpm	HFNC is associated with fewer desaturation episodes and hypoxia. There is no difference in the incidence of hypercarbia. Post-hoc analyses showed that patients with COPD who received HFNC had a significantly higher incidence of hypercarbia without differences in hypoxemia.
Thiruvenkatarajan, 2021 [18]	RTC 132	Adults with moderate to high risk for hypoxemia: BMI > 30 or OSA or ASA classification of III–IV undergoing ERCP.	Propofol, Fentanyl	HFNC: Flow 30–60 lpm and FiO_2_ 100% vs. COT through nasal cannula at 4 lpm + 4 lpm through mouthguard.	HFNC vs. COT did not significantly decrease hypoxemia, hypercarbia, the need for airway interventions, the requirement of a chin lift/jaw thrust, nasopharyngeal airway insertion, bag-mask ventilation, or OTI.
Zhang, 2022 [19]	RCT 369	Elderly patients; ASA classification of I–II; and BMI < 30 kg/m^2^ undergoing gastroscopy	Propofol	HFNC: Flow 30 lpm and FiO_2_ at 50% (H50) or 100% (H100) vs. COT at 8 lpm	The incidence of hypoxia was lower in both the H50 and H100 groups than in the COT group. No significant differences were seen in the incidence of hypoxia between the H50 and H100 groups.
Sawase, 2023 [20]	RCT 75	Adult patients (20–82 years) undergoing ERCP	Midazolam, Pethidine, hydrochloride	HFNC: Flow 40–60 lpm and FiO_2_ 21% vs. COT at 1–2 lpm.	HFNC with room air vs. COT did not reduce marked hypercapnia during ERCP under sedation. There was no significant difference in the occurrence of hypoxemia between the HFNC group and the COT group.
Kim, 2021 [21]	RCT 72	Adults with moderate to high risk for hypoxemia are undergoing ERCP in the prone position.	Propofol, Fentanyl	HFNC: Flow 50 lpm and FiO_2_ 100% vs. COT at 5 lpm	HFNC provided a better nadir SpO_2_ level under sedation and less procedural interruption.
Lin, 2023 [22]	SC 294	Adults undergoing anatomical resections, lymph node biopsy, and staging through uniportal VATS	Fentanyl, Propofol, Desflurane or Sevoflurane are inhaled	Airway management was performed with i-gel (Intersurgical Ltd.) or HFNC Optiflow (Fisher & Paykel).	HFNC had a significantly higher desaturation event rate, lower nadir SpO2, and longer hospitalisation compared to the i-gel group. However, propensity score matching analysis revealed no significant between-group difference in the desaturation rate.
Flach, 2019 [23]	SC 21	Transoral laser microsurgery for subglottic stenosis, cordectomy, excision of a laryngeal papilloma, or vocal cord lesion.	Propofol, Remifentanil	HFNC: Flow 70 lpm and FiO_2_ 100%.	No intra- or immediate post-operative complications were recorded, and adequate surgical access was achieved. No airway fires or similar adverse events, such as flaring, were mentioned.
Ke, 2020 [24]	SC 160	Adults undergoing resection VATS	Propofol, Fentanyl, Midazolam.	HFNC: Flow 50 lpm and FiO_2_ 100 vs. OTI (double-lumen endotracheal tube).	OTI had a significantly longer mean induction and operative time, suffered greater intraoperative blood loss, had longer postoperative hospital stays, and had an increased chest tube retention time vs. HFNC.
Benninger, 2021 [25]	SC 53	Patients with subglottic stenosis, vocal cord lesions, and vocal cord paralysis through microlaryngoscopy.	Propofol, Remifentanil	THRIVE: Flow 70 lpm and FiO_2_ 100%.	The median apnea time was 16 min, the median end tidal CO_2_ was 50 mmHg, and the median minimum SpO_2_ was 95%. Six cases required supplementation of THRIVE with OTI for sustained oxygen desaturation.
Lai, 2018 [26]	SC 256	VATS for lung biopsy, wedge resection, segmentectomy, lobectomy, mediastinal tumour excision, and bullectomy.	Propofol to achieve BIS level 40–60.	THRIVE: Flow 20 lpm and FiO_2_ indicated before anaesthesia vs. COT through conventional O_2_ mask	Postoperative temperatures were significantly higher in patients using THRIVE vs. COT. Significantly less intraoperative temperature decrease was shown in the THRIVE group.

RCT: randomised control trial. FOB: fiberoptic bronchoscopy. BAL: bronchoalveolar lavage. HFNC: High Flow Nasal Cannula. Lpm: litres per minute. Tª: temperature. COT: conventional oxygen therapy. EBUS: endobronchial ultrasound. SC: single centre. NIV: non-invasive ventilation. PEEP: Positive end-expiratory pressure. IPAP: inspiratory positive airway pressure. ERCP: endoscopic retrograde cholangiopancreatography. ASA: American Society of Anesthesiologists physical status class. BMI: body mass index. EGD: Esophagogastroduodenoscopy. COPD: chronic obstructive pulmonary disease. OSA: Obstructive sleep apnoea. OTI: orotracheal intubation. VATS: video-assisted thoracic surgery. THRIVE: transnasal humidified rapid-insufflation ventilatory exchange. BIS: frontal bispectral index.
